# Numerical Investigation on the Anti-Angiogenic Therapy-Induced Normalization in Solid Tumors

**DOI:** 10.3390/pharmaceutics14020363

**Published:** 2022-02-05

**Authors:** Mahya Mohammadi, Cyrus Aghanajafi, M. Soltani, Kaamran Raahemifar

**Affiliations:** 1 Department of Mechanical Engineering, K. N. Toosi University of Technology, Tehran 19967-15433, Iran; mahya.mohammadi@email.kntu.ac.ir (M.M.); aghanajafi@kntu.ac.ir (C.A.); 2 Department of Applied Mathematics, University of Waterloo, Waterloo, ON N2L 3G1, Canada; 3 Department of Electrical and Computer Engineering, University of Waterloo, Waterloo, ON N2L 3G1, Canada; 4 Advanced Bioengineering Initiative Center, Multidisciplinary International Complex, K. N. Toosi University of Technology, Tehran 14176-14411, Iran; 5 Centre for Biotechnology and Bioengineering (CBB), University of Waterloo, Waterloo, ON N2L 3G1, Canada; 6 Data Science and Artificial Intelligence Program, College of Information Sciences and Technology (IST), Penn State University, State College, PA 16801, USA; kraahemi@gmail.com; 7 School of Optometry and Vision Science, Faculty of Science, University of Waterloo, 200 University Ave W, Waterloo, ON N2L 3G1, Canada; 8 Department of Chemical Engineering, University of Waterloo, 200 University Avenue W, Waterloo, ON N2L 3G1, Canada

**Keywords:** normalization, anti-angiogenic therapy, non-homogeneous solid tumor, necrotic area, reservoir behavior

## Abstract

This study numerically analyzes the fluid flow and solute transport in a solid tumor to comprehensively examine the consequence of normalization induced by anti-angiogenic therapy on drug delivery. The current study leads to a more accurate model in comparison to previous research, as it incorporates a non-homogeneous real-human solid tumor including necrotic, semi-necrotic, and well-vascularized regions. Additionally, the model considers the effects of concurrently chemotherapeutic agents (three macromolecules of IgG, $F{\left(ab^{\prime }\right)}_{2}$, and $F(ab^{\prime })$) and different normalization intensities in various tumor sizes. Examining the long-term influence of normalization on the quality of drug uptake by necrotic area is another contribution of the present study. Results show that normalization decreases the interstitial fluid pressure (IFP) and spreads the pressure gradient and non-zero interstitial fluid velocity (IFV) into inner areas. Subsequently, wash-out of the drug from the tumor periphery is decreased. It is also demonstrated that normalization can improve the distribution of solute concentration in the interstitium. The efficiency of normalization is introduced as a function of the time course of perfusion, which depends on the tumor size, drug type, as well as normalization intensity, and consequently on the dominant mechanism of drug delivery. It is suggested to accompany anti-angiogenic therapy by $F(ab^{\prime })$ in large tumor size ($R_{eq}=2.79\;\;\mathrm{cm}$) to improve reservoir behavior benefit from normalization. However, IgG is proposed as the better option in the small tumor ($R_{eq}=0.46\;\;\mathrm{cm}$), in which normalization finds the opportunity of enhancing uniformity of IgG average exposure by 22%. This study could provide a perspective for preclinical and clinical trials on how to take advantage of normalization, as an adjuvant treatment, in improving drug delivery into a non-homogeneous solid tumor.

## 1. Introduction

Mathematical modeling has a significant role in the diagnosis and treatment of cancer [1,2,3,4]. Cancer has a multi-scale nature spanning from intracellular to tissue, in which mathematical modeling is used in all scales [5]. There exist recent studies [5,6,7,8,9,10,11,12,13] that applied mathematical modeling in different scales to simulate the various processes in the tumor microenvironment and its diagnosis and treatment. Recently, Hadjicharalambous et al. [14] provided a review on in silico modeling of tumor perfusion, angiogenesis, drug delivery, and studies that took advantage of clinical data. The present research studies drug delivery in cancer treatment.

Even though the most important treatment is surgery to remove the tumor [15], chemotherapy is an extensively used tool in cancer treatment [16]. The quality of chemotherapy depends on the efficient delivery of therapeutic agents into the cancerous zones [17]. Numerous studies have been performed to investigate drug delivery by mathematical modeling. In fundamental studies [18,19,20], Baxter and Jain developed a macroscopic mathematical model by considering the tumor microenvironment as a porous medium. They studied the effect of interstitial fluid properties, heterogeneous perfusion of the tumor, presence of lymphatics in the tumor, binding, and metabolism on macromolecule distribution in the extracellular matrix (ECM). They found high IFP as the main barrier to drug distribution uniformity. Moreover, it was concluded that the presence of lymphatics in the tumor site reduced the concentration amount. In the third phase of the study of Baxter and Jain, the important role of binding on macromolecule distribution was derived. Other studies have been conducted from a macroscopic point of view, such as [21,22], in which drug transport was considered by fluid flow. Two parameters, i.e., the critical radius of tumor and necrotic area, were introduced as effective parameters of drug delivery. In the next step of the previous research, the effect of tumor shape and size was studied by adding solute transport equations to the mathematical model [23]. The results showed that drug concentration in the prolate shape of the tumor had the highest value, which is due to the non-uniform distribution of IFP in this shape, unlike other forms. Steuperaert et al. [24] studied intraperitoneal chemotherapy by solving fluid flow and solute transport equations in tissue scale, then expanded their model to consider the real shape of the tumor and non-uniform interstitial properties [25]. Multi-scale research [8,9,11,12] has been developed to study drug delivery by considering more details such as image-based microvascular network, intravascular flow, and the relationship between the flow of capillary network and interstitium. Drug delivery was studied in a comprehensive multi-scale model, in which the tumor-induced capillary network was performed by a mathematical model [26]. In the study of Moradi Kashkooli and Soltani [27], the importance of alternative chemotherapy strategies—metronomic chemotherapy and chemo-switching—in consecutive treatments were addressed compared to the conventional approach, which is based on maximum tolerated dose, through a numerical model on a real image of solid tumor. They proposed an appropriate computational framework to evaluate and improve the treatment efficacy of solid tumors.

The characteristic of solid tumors such as high IFP and an abnormally tortuous capillary network make the delivery and efficacy of therapeutic agents difficult [28]. The methods to overcome it are a subject of interest amongst researchers [16]. One of these methods is anti-angiogenic treatment [29], which, although it does not show much ability alone, in combination with other treatments such as chemotherapy, causes long-term clinical benefit or survival [30,31]. Clinical studies [32,33] have shown that the combination of the anti-angiogenic drug with a chemotherapy drug has more positive effects than using chemotherapy alone in reducing tumor size, mean vessel diameter, and irregular morphology of vessels, and in improving survival effect. Anti-angiogenic drugs have also been shown to improve drug delivery efficacy and deeper drug penetration [34,35,36]. In recent reviews [37,38,39], normalization induced by anti-angiogenesis in combination with chemotherapy was introduced as a promising strategy in cancer treatment. Few numerical studies have been performed on drug delivery under the influence of anti-angiogenesis-induced vascular normalization. One of the most important and basic of these studies is [40], which investigated the effect of normalization in a homogeneous avascular tumor by simulating the interstitial fluid flow. The major finding of Jain and his colleagues’ research is that IFP decreased after normalization. Mohammadi et al. [41] expanded the mathematical framework of [40] to consider the solute transport equation. Their results showed that drug delivery into the single homogeneous tumor nodule improved by normalization. Zhan [42] investigated the efficacy of cytotoxic drug delivery into the tumor based on a real brain tumor under the anti-angiogenic effect of Bevacizumab. The author used a diffusion–convection equation to describe Bevacizumab bioavailability in tissue. The results showed enhancement of drug delivery by Bevacizumab administration. Other studies [43,44] followed up the effect of anti-angiogenic therapy on therapeutic agent transfer into the tumor site by developing governing partial differential equations. Sweeney et al. [45] mimicked the vascular normalization by modifying the parameters of interstitial and intravascular flow in a tumor with a real image-based capillary network. Wu et al. [46] and Moath and Xiao [47] studied the normalization by pruning the microvascular network produced by mathematical modeling. Stylianopoulos and Jain [48] developed a mathematical framework to consider the normalization from another point of view, in which decreasing the vessel diameter and pruning the microvasculature network were considered as anti-angiogenic therapy consequences. They demonstrated that vessels with more permeability and less compressibility were affected more by normalization.

Due to the necessity of opening a horizon in how the quality of drug delivery into the solid tumor is affected by anti-angiogenesis-induced normalization (AAIN), the present study addresses this issue by developing a numerical framework to model fluid flow and solute transport in the tumor interstitium. A more realistic physiological model of ECM is provided in this research for the first time by considering the computational domain based on the cross-sectional view of a real-human tumor, which contains the necrotic core, semi-necrotic region, and well-vascularized area. Simultaneous study of different effective parameters in the investigation of the AAIN in connection with the drug delivery is in need as the resolution of the AAIN function is not accurate without considering all the significant factors. Moreover, the behavior of therapeutic agent distribution in the necrotic core over long periods under the influence of AAIN has not been studied before, to the best of the authors’ knowledge. To fill these gaps, various efficacious aspects, i.e., tumor size, therapeutic agent type, post-injection time, and normalization intensity, which control the efficiency of AAIN on drug delivery, are studied in the present research. Additionally, the reservoir behavior of the necrotic area is analyzed under the effect of AAIN.

## 2. Materials and Methods

In the current study, a real solid tumor is considered to investigate the drug delivery under the influence of different intensities of AAIN. This section consists of the computational geometry, schematic view of AAIN function, governing equations, numerical solution details, and baseline value of parameters.

### 2.1. Computational Geometry

**Figure 1:**
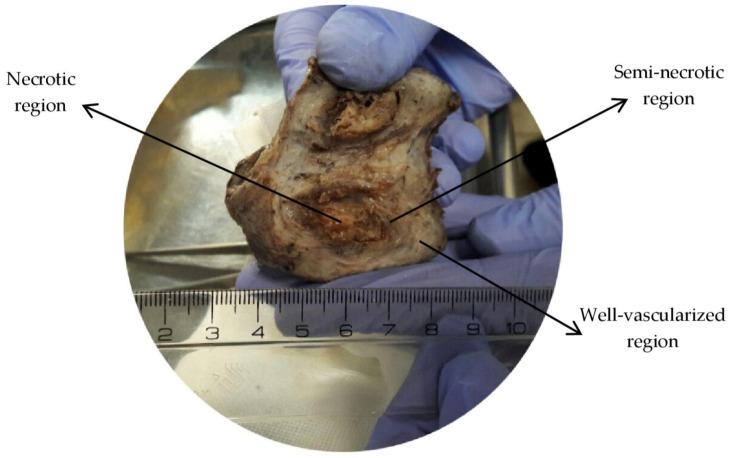
Geometry of studied tumor with different parts.

**Figure 2:**
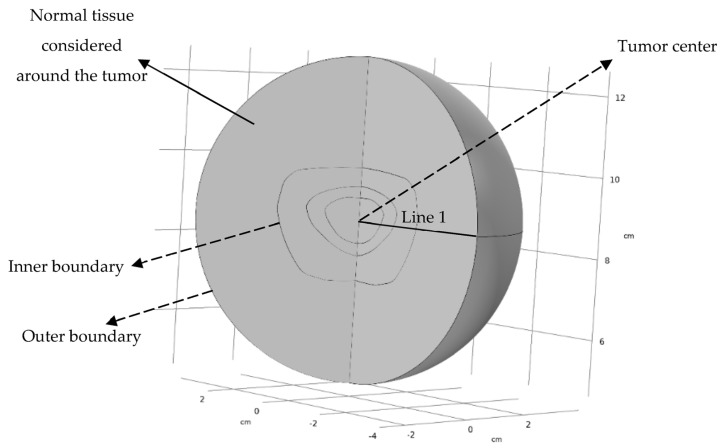
Computational geometry and boundaries. Results are drawn along line 1.

Figure 1 shows the cross-sectional view of the real tumor studied in this research. Different parts of the tumor are illustrated in this figure. Due to the availability of one image of the tumor and the approximate symmetry of the cross-sectional view, half of this view is drawn, and the computational domain is considered axisymmetric. Figure 2 shows the computational geometry around which normal tissue is considered. Line 1, shown in Figure 2, is used to draw IFP, IFV, and concentration profiles along it.

### 2.2. Schematic View of AAIN Function

**Figure 3:**
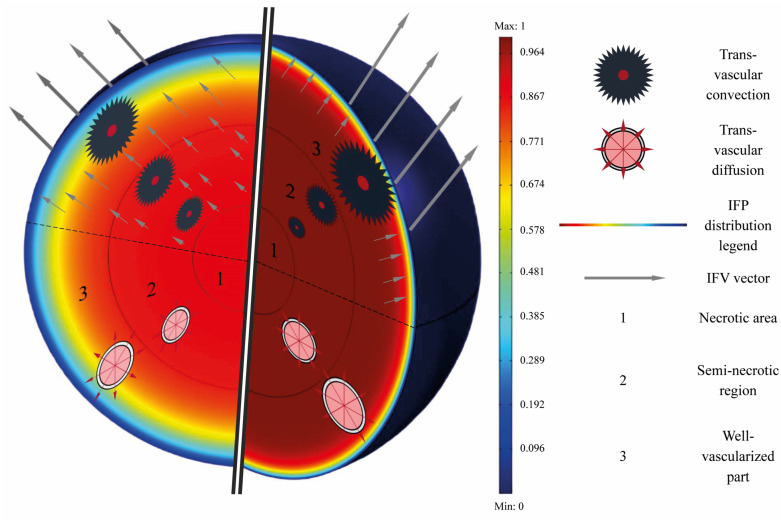
Cross-sectional schematic of a non-homogeneous solid tumor before and after the anti-angiogenic therapy.

Figure 3 provides a perspective of the performance of AAIN. As illustrated in this figure and discussed in the following, anti-angiogenic therapy normalizes the tumor vasculature, which results in (1) decreasing the IFP in the interstitium, (2) establishing the IFP gradient and non-zero IFV in the areas far from the tumor periphery, (3) reducing the IFV at the tumor margin, (4) decreasing therapeutic agents oozing from the boundary, (5) improving the trans-vascular convection mechanism of solute transport, and (6) modifying the trans-vascular diffusion mechanism of solute transport.

### 2.3. Governing Equations

In this investigation, fluid flow and solute transport are simulated to study the drug delivery into the solid tumor with a macroscopic model.

#### 2.3.1. Fluid Flow Mathematical Model

From a macroscopic point of view, the microscopic variations are average [18]. The simplified momentum equation governing the fluid flow in the interstitium, as a porous medium, is expressed by Darcy’s equation as follows [21,22]:

(1) $${\vec{V}}_{i}=-k\nabla P_{i}$$

in which ${\vec{V}}_{i}$, k , and $P_{i}\;$ are IFV, interstitium hydraulic conductivity, and IFP, respectively. k  is considered to be constant in this study.

The continuity equation for the incompressible fluid in the steady-state considering the source and sink terms that exist in biological tissues is as follows:

(2) $$\nabla .{\vec{V}}_{i}=\phi_{B}-\phi_{L}$$

$\phi_{B}$ and $\phi_{L}$ show the rate of fluid flow from the blood vessels to the interstitium and from the interstitium to the lymphatic vessels, respectively [21]. $\phi_{B}$ and $\phi_{L}$ are reported in Table 1. Due to the lack of an efficient lymphatic system in tumor tissue, $\phi_{L}$ is considered only in normal tissue with a uniform distribution [26].

Combining Equations (1) and (2) results in:

(3) $$-k\nabla^{2}P_{i}=\phi_{B}-\phi_{L}$$

#### 2.3.2. Solute Transport Mathematical Model

The governing equation of solute transport in the porous media involves two mechanisms of diffusion and convection [50]. Mass conservation by using Fick’s second law is determined as follows to define the behavior of solute transport [11,26]:

(4) $$\frac{\partial C_{i}}{\partial t}+\nabla \cdot \vec{J}=0$$

where $C_{i}$ represents the concentration of solute in the interstitium. $\vec{J}$ is the mass flux of the solute.

The diffusion mechanism of the solute transport in the biological tissues is obtained from Fick’s first law. The convection mechanism is calculated by multiplying the concentration by the IFV. Therefore, $\vec{J}$ is as follows [11]:

(5) $$\vec{J}=-D_{eff}\nabla C_{i}+{\vec{V}}_{i}C_{i}$$

The final equation governing the solute transport, taking into account the source and sink terms of biological tissues with the constant diffusion coefficient ($D_{eff}$), can be determined with the following equation [11,26]:

(6) $$\frac{\partial C_{i}}{\partial t}=D_{eff}\nabla^{2}C_{i}-\nabla \cdot ({\vec{V}}_{i}C_{i})+\varphi_{B}-\varphi_{L}$$

$\varphi_{B}$ and $\varphi_{L}$ indicate the rate of solute transfer per unit volume from the blood vessels to the interstitial space and from the interstitial space to the lymphatic vessels, respectively. Patlak’s model is used to calculate the transition rate of the solute from the blood vessel walls [51]. $\varphi_{L}$ is defined by a function that has a uniform distribution only in normal tissue [23]. $\varphi_{B}$ and $\varphi_{L}$ are reported in Table 1. It is assumed that solute transport does not affect the interstitial fluid density and interstitial fluid flow. So, equations for fluid flow analysis are calculated independent of that of solute transport analysis.

### 2.4. Numerical Solution Details

This section consists of the description of the mesh-independent solution, boundary conditions, and numerical modeling procedure.

#### 2.4.1. Mesh Independent Solution

For checking the independence of the solution from the mesh size, results of the fluid flow and solute transport analyses (IFP, IFV, and $C_{i}$) with different grids are compared. The final size of meshing of the computational domain is selected so that the percentage of change in the results between the last two mesh sizes is negligible. The final mesh of the original tumor size consists of 19,714 elements. This process is carried out for the other studied sizes.

#### 2.4.2. Boundary Conditions

Boundary conditions (BCs) of analyses of fluid flow and solute transport are reported in Table 2. The different parts of the tumor in which the BCs are defined can be seen in Figure 2. Due to the symmetry, the BC of the tumor center is no flux for both fluid flow and solute transport analyses [15]. The BC of the inner boundary is such that the pressure and velocity in fluid flow analysis and the concentration and concentration flux in solute transport analysis are continuous [9,11]. At the outer boundary, the pressure is equal to the surrounding pressure [15], which is considered to be 0 Pa in the present study. The open BC is applied for solute transport analysis to model the mass transfer across the outer boundary wherein each convective inflow and outflow can occur [52].

#### 2.4.3. Numerical Modeling Procedure

**Figure 4:**
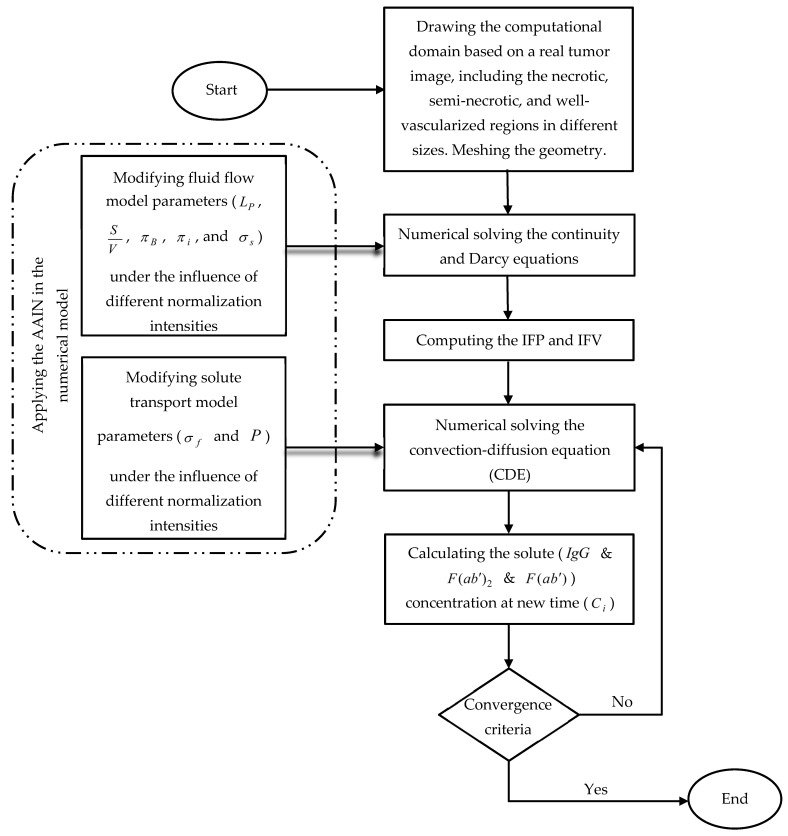
The flowchart of the numerical modeling procedure.

Steady fluid flow equation and transient solute transport equation are solved numerically with finite element method (FEM) using COMSOL Multi-physics ^®^ Software Version 5.3a. The discretization method of fluid flow and solute transport models are quadratic and linear, respectively. Newton’s method becomes implemented for solving equations. The criteria for convergence are to decrease the residuals to be $10^{-6}$. The flowchart of the simulation strategy is shown in Figure 4.

### 2.5. Baseline Value of Parameters

The parameter values are described in this section in different types of tissue. Vascular hydraulic permeability, $L_{P}$, was calculated to be $3.6\times 10^{-8}\;\mathrm{cm}/s/\mathrm{mmHg}$ in the skeletal muscle of normal tissues of rats [53]. This value is chosen in the present study for normal tissue. $L_{P}$ in tumor tissue is considered to be 7.8 times that of normal tissue, according to the measurements by Gerlowski and Jain [18]. Jain et al. [40] mentioned that treatment by anti-VEGFR2 antibody causes a 5-fold decrease in $L_{P}$ of the tumor tissue. This rate of decrease is used in the present study for normalized tissue.

The value of hydraulic conductivity of the interstitium, k , is assumed to be the same in tumor and normal tissues in this study based on [40]. The effect of AAIN on k  is not known to the best of our knowledge. k  in normalized tissue is assumed to be the same as the tumor one.

The value of $\frac{S}{V}$, vessel surface density, has a large variability across different types of tumor and even within the individual tumor. The value of $\frac{S}{V}$ for different tissue types ranges from 50 to 570 1/cm [40,54]. $\frac{S}{V}$ is chosen to be 70 and 200 1/cm for normal and tumor tissue, respectively, based on previous studies [22,23]. Some in vivo studies [55,56] showed a decrease in the permeability surface area product after anti-angiogenic therapy by Anti-VEGF antibody. Therefore, in the present modeling, $\frac{S}{V}$ is considered to be 116 1/cm after normalization.

$P_{B}$ fell in the range of 5.3–34  mmHg in different types of tissue [40]. $P_{B}$ was considered equal to 15.6 mmHg for both normal and tumor tissue in the previous studies [18,22,23]. Measurement of microvascular pressure in MCaIV tumors by Jain’s group showed that the change in $P_{B}$ before and after anti-VEGFR2 therapy is negligible [57]. $P_{B}$ is considered to be 15.6 mmHg for all types of tissue in this study. $\pi_{B}$ and $\pi_{i}$ were also measured before and after anti-VEGFR2 therapy [57]. The value of these parameters is considered the same as the value reported in [40].

$\sigma_{s}$, the osmotic reflection factor, was measured for albumin in subcutaneous normal tissue [58]. $\sigma_{s}$ was not reported for tumors. For tumor tissue, $\sigma_{s}$ can be approximated using $\sigma_{s}={\left[1-{\left(1-\lambda \right)}^{2}\right]}^{2}$ where $\lambda =\frac{\mathrm{Solute}\;\;\mathrm{radius}}{\mathrm{Pore}\;\;\mathrm{radius}}$ [59,60]. Albumin was considered as a sphere with a 3.5 nm radius in Bovine serum [54,61]. The diameter of the vascular pore was chosen to be 1.5 μm. $\sigma_{s}$ is calculated for normalized tissue with the same formula of tumor tissue and a one-fifth reduction in pore size of vessels induced by anti-angiogenic therapy [40].

$\sigma_{f}$ shows the osmotic reflection coefficient. Baxter and Jain [18] used the data of [62] for defining the value of $\sigma_{f}$. Covell et al. [62] noted that the metabolism of the antibody was specified by non-tumor tissue. The value of $\sigma_{f}$ for different tissue types is estimated based on the spherical solute–cylindrical pore model [59,60] and listed in Table 3.

$D_{eff}$ and $P_{eff}$ represent the effective interstitial diffusion coefficient and effective microvascular network permeability coefficient, respectively. In this study, the value of $D_{eff}$ and $P_{eff}$ is defined in normal and tumor tissue for IgG, $F{\left(ab^{\prime }\right)}_{2}$, and $F(ab^{\prime })$ based on the study of Gerlowski and Jain [63]. The effect of normalization induced by anti-angiogenic therapy on $D_{eff}$ is not known, to the best of our knowledge. It is assumed that $D_{eff}$ in tumor tissue does not change after vascular normalization. The diffusive microvessel permeability coefficient, P, is considered to be 10% of $P_{eff}$ [23]. A decrease of 68% in vascular permeability ($P\frac{S}{V}$ [64]) was reported after treatment by Bevacizumab [65]. The baseline values of interstitial fluid flow and solute transport properties are summarized in Table 3. Transport properties of other normalization intensities are calculated between baseline values.

## 3. Validation of Numerical Model

**Figure 5:**
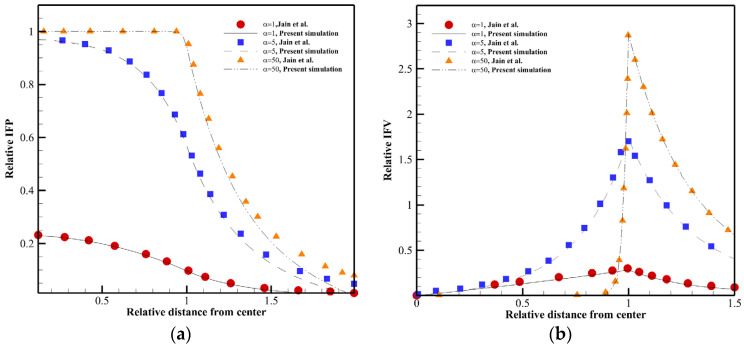
Comparison between the result of the present model and literature [40]. A tumor (*R* = 0.4 cm) embedded in the normal tissue is studied in this figure. (**a**) Distribution of the relative IFP. (**b**) Distribution of the relative IFV.

**Figure 6:**
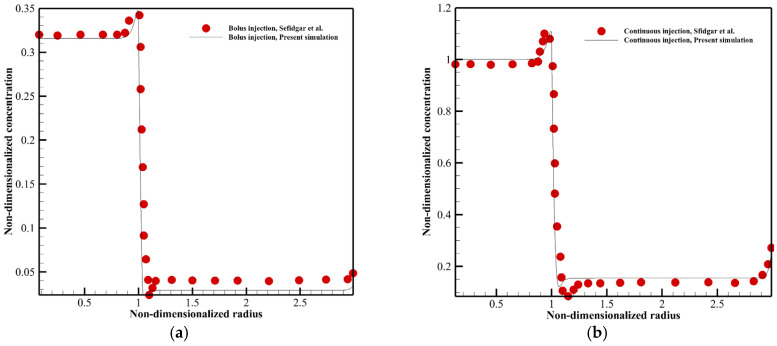
Comparison between the result of the present model and literature [23]. A tumor (*R* = 1 cm) surrounded by normal tissue is studied in this figure. (**a**) Interstitial $F{\left(ab^{\prime }\right)}_{2}$ concentration in 8 h post-injection with bolus injection. (**b**) Interstitial $F{\left(ab^{\prime }\right)}_{2}$ concentration in 72 h post-injection with continuous injection.

**Figure 7:**
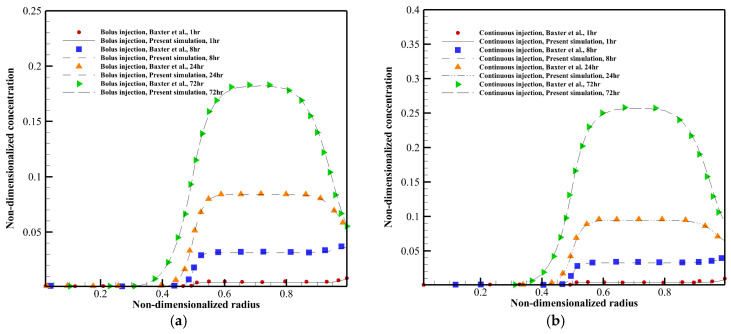
Comparison between the result of the present model and literature [19]. A tumor (*R* = 1 cm) with a 0.5 ratio of the necrotic radius to the tumor radius is investigated in this figure. (**a**) Interstitial IgG concentration with bolus injection. (**b**) Interstitial IgG concentration with continuous injection.

Different case studies of the literature [19,23,40] are duplicated numerically to verify the present model. Figure 5 shows the fluid flow properties of a homogeneous solid tumor embedded within normal tissue. Interstitial drug concentration in a homogeneous solid tumor surrounded by normal tissue is seen for continuous and bolus injections in Figure 6a,b. Figure 7a,b illustrates the distribution of the therapeutic agent in a tumor with the necrotic core for continuous and bolus injections.

As seen in Figure 5, the IFP is uniformly high throughout the tumor in α=50. There is a pressure gradient and then non-zero IFV only in the boundary between tumor and normal tissues. α with the equation of $\alpha =R\sqrt{\frac{L_{p}S}{kV}}$ shows the rate of transport across the vessel wall to the rate through the interstitium [40]. Changing α causes modification in IFP and IFV behavior. Figure 6 shows that the procedure of $F{\left(ab^{\prime }\right)}_{2}$ distribution is the same in bolus injection (8 h post-injection) and continuous injection (72 h post-injection), but the drug exposure is greater in continuous injection. As shown in Figure 7, the solute concentration in tumor tissue has non-uniform distribution due to considering the necrotic area, unlike Figure 6.

According to Figure 5, Figure 6 and Figure 7, the agreement between the results of this study and those of the literature is very good that shows the accuracy of the numerical method used in the present study in predicting drug delivery into solid tumors.

## 4. Results and Discussion

In the present study, the behavior of fluid flow and drug distribution in a real-solid-human-non-homogeneous tumor with normal tissue around it are studied to investigate the effect of intensity of AAIN by solving the continuity, Darcy, and CDEs. IFP, IFV, and solute concentration distribution at different normalization intensities are analyzed below. The parameters of average solute concentration distribution (ASCD) and its deviation (DASCD) are introduced and investigated to demonstrate the quality of drug delivery into the tumor tissue and its distribution uniformity. ASCD and DASCD are defined by Equations (S1) and (S2). Different tumor sizes and different drugs are considered for a closer look and a more accurate deduction on the effect of normalization intensity.

In this study, α is defined as $\alpha =R_{eq}\sqrt{\frac{L_{p}S}{kV}}$. $R_{eq}$ is the radius of a spherical tumor with the same volume of tumor of the present study [22]. $R_{eq}$ is equal to approximately 1.86 cm.

The spatial characteristics of the different parts of the tumor on line 1 in Figure 2 are as follows:

$$\left\{ \begin{array}{l} 0\leq \frac{r}{R_{eq}}\leq 0.4192903124\;\;\;\;\;\;\;\;\;\;\;\;\;\;\;\;\;\;\;\mathrm{Necrotic}\;\mathrm{region}\\ 0.4192903124\;\leq \frac{r}{R_{eq}}\leq 0.6572532333\;\;\;\mathrm{Semi}-\mathrm{necrotic}\;\mathrm{region}\\ 0.6572532333\;\leq \frac{r}{R_{eq}}\leq 1.0646712645\;\;\;\mathrm{Well}-\mathrm{vascularized}\;\mathrm{region} \end{array}\right.$$

### 4.1. Fluid Flow Analysis

**Figure 8:**
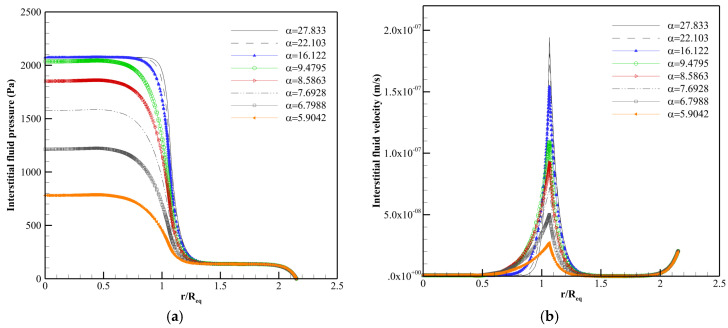
(**a**) IFP distribution along line 1. (**b**) IFV distribution along line 1.

According to Equations (1) and (2), IFP has a significant effect on different mechanisms of drug delivery. High IFP in the tumor tissue and its sudden decrease at the tumor margin are some main barriers to effective drug delivery [9,21,22]. As shown in Figure 8a, IFP in the untreated tumor (α=27.833) has its maximum value, and it decreases with the highest amount of slope at the margin of tumor and normal tissues. IFP in the tumor tissue is reduced by anti-angiogenesis therapy, a phenomenon that has been reported in clinical and preclinical research [57,67,68,69]. The greater the intensity of normalization, the greater the IFP drop.

The pressure gradient is established in semi-necrotic and well-vascularized regions due to the normalization. IFP and $P_{eff}$ in most parts of the tumor tissue are almost equal in high values of α, which causes a lack of net flow exchange and subsequently a high uniform IFP. AAIN changes this behavior due to affecting the parameters of the blood source ($\phi_{B}$ in Equation (3)). Forasmuch as there is well-vessel density in the well-vascularized region, the pressure gradient induced by anti-angiogenesis is considerable in this area. Normalization cannot cause a pressure gradient in the tumor necrotic core because there are no effective blood vessels in this area.

In high values of α, IFV has a non-zero value only in the tumor tissue boundary, which causes outward convection flux. The behavior of the IFV profile depends on the pressure gradient according to Darcy’s law. IFV becomes non-zero within the tumor (in semi-necrotic and well-vascularized regions) under the effect of vascular normalization, as seen in Figure 8b. It is seen that the amount of IFV is reduced at the tumor boundary after anti-angiogenic therapy. This is due to the fact that normalization causes a less steep in IFP gradient.

Areas where there is a pressure gradient and subsequently non-zero IFV shift to the inner parts of the tumor tissue by decreasing α from 27.833 to 9.4795. A further decrease in α cannot cause convective flow in the more internal areas of the tumor. However, the number of changes of IFP and IFV in the range of 5.9042<α<9.4795 is more severe. It means that the effectiveness of normalization depends on the properties of the baseline parameters. As shown in Figure 8b, the value of IFV is low in areas other than the boundary, which indicates the small effect of convection in these zones.

### 4.2. Solute Transport Analysis

The effects of normalization intensity on the concentration distribution of IgG, $F{\left(ab^{\prime }\right)}_{2}$, and $F(ab^{\prime })$ are investigated. A single bolus injection is considered with a function that the concentration of plasma solute decreases exponentially with time. The half-life of these macromolecules is different from each other. Simulation for each drug is considered until the ASCD in the necrotic core (ASCDNE) reaches one-tenth of its maximum value. This time for different antibodies in different sizes of tumor is as follows:

$$R_{eq}=0.46\;\mathrm{cm}\;\;\;\left\{ \begin{array}{l} F(ab^{\prime }):179,640s\;(2.08\;\mathrm{days})\\ F{\left(ab^{\prime }\right)}_{2}:\;488,520s\;(5.65\;\mathrm{days})\\ IgG:\;1,504,800s\;(17.41\;\mathrm{days})\; \end{array}\right.$$

$$R_{eq}≃0.93\;\mathrm{cm}\;\;\;\left\{ \begin{array}{l} F(ab^{\prime }):403,920s\;(4.675\;\mathrm{days})\\ F{\left(ab^{\prime }\right)}_{2}:\;1,297,100s\;(15\;\mathrm{days})\\ IgG:\;3,474,000s\;(40.2\;\mathrm{days})\; \end{array}\right.$$

$$R_{eq}≃1.86\;\mathrm{cm}\;(Original\;size)\;\;\;\left\{ \begin{array}{l} F(ab^{\prime }):761,760s\;(8.8\;\mathrm{days})\\ F{\left(ab^{\prime }\right)}_{2}:\;2,791,100s\;(32.3\;\mathrm{days})\\ IgG:\;7,768,800s\;(89.9\;\mathrm{days})\; \end{array}\right.$$

$$R_{eq}=2.79\;cm\;\;\;\left\{ \begin{array}{l} F(ab^{\prime }):974,160s\;(11.275\;\mathrm{days})\\ F{\left(ab^{\prime }\right)}_{2}:\;3,988,100s\;(46.16\;\mathrm{days})\\ IgG:\;12,017,000s\;(139\;\mathrm{days})\; \end{array}\right.$$

**Figure 9:**
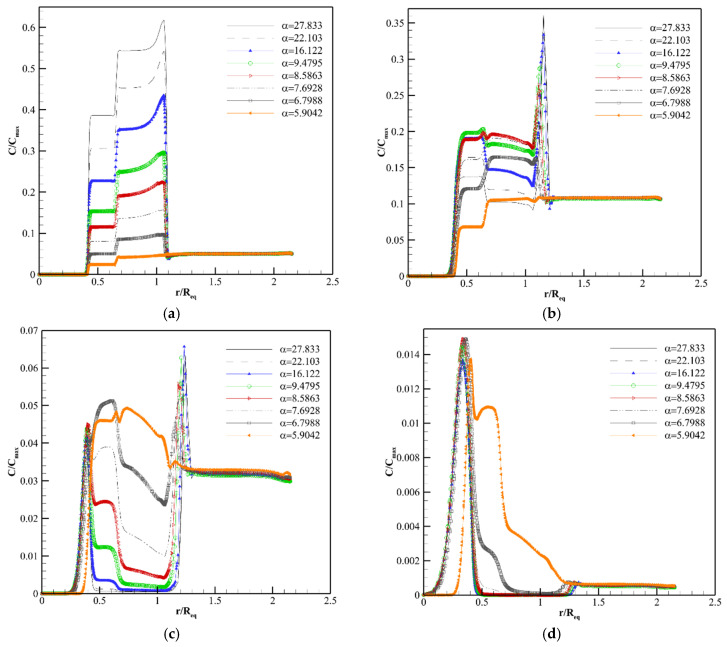
Distribution of $F(ab^{\prime })$ concentration along line 1. (**a**) 1 h post-injection. (**b**) 8 h post-injection. (**c**) 24 h post-injection. (**d**) 72 h post-injection.

**Figure 10:**
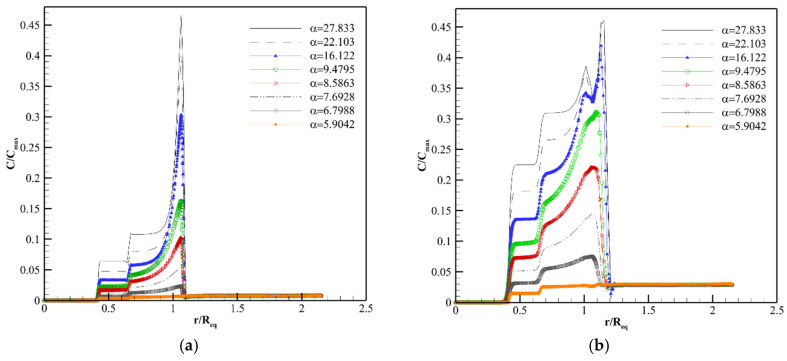

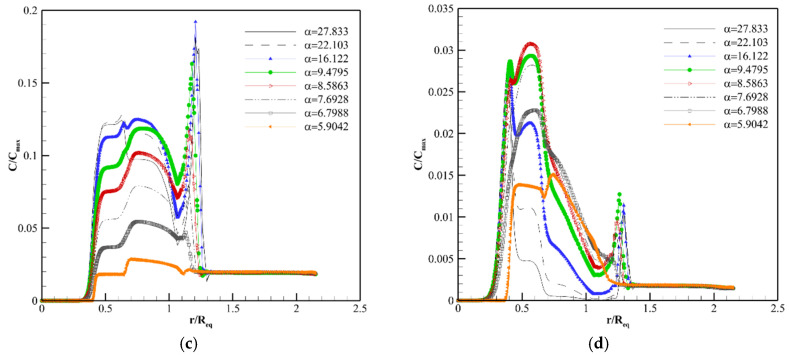
Distribution of $F{\left(ab^{\prime }\right)}_{2}$ concentration along line 1. (**a**) 1 h post-injection. (**b**) 8 h post-injection. (**c**) 24 h post-injection. (**d**) 72 h post-injection.

**Figure 11:**
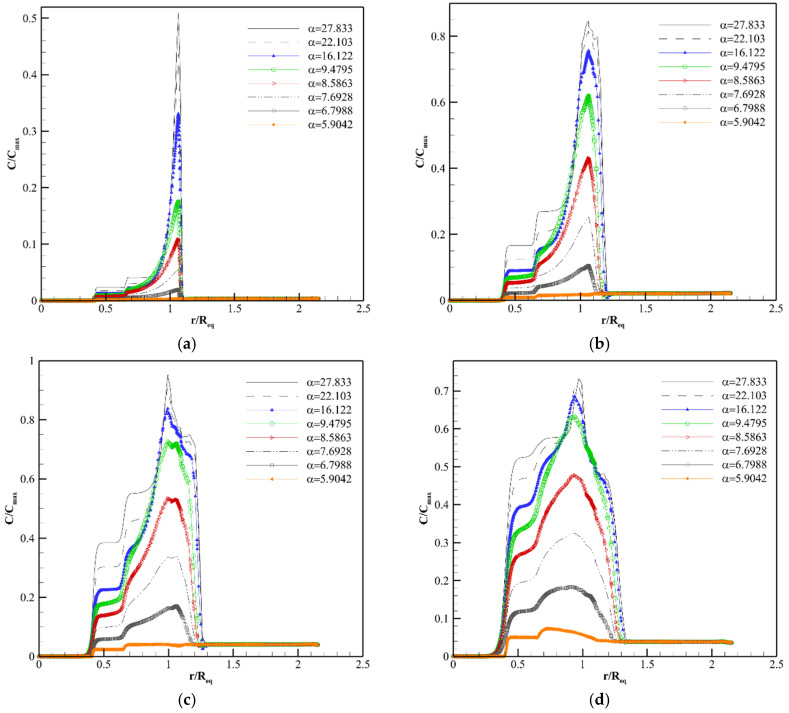
Distribution of IgG concentration along line 1. (**a**) 1 h post-injection. (**b**) 8 h post-injection. (**c**) 24 h post-injection. (**d**) 72 h post-injection.

Figure 9, Figure 10 and Figure 11, Figures S1 and S2 show the concentration distribution of $F(ab^{\prime })$, $F{\left(ab^{\prime }\right)}_{2}$, and IgG along line 1 in $R_{eq}=1.86\;\mathrm{cm}$ at different post-injection times in various αs. As seen in these figures, there is a non-uniform concentration distribution in all macromolecules because in the present work, unlike previous studies [18,22,23,40,64], the tumor is not homogeneous and different parts of the tumor are considered.

There is no blood source in the necrotic region. Additionally, the IFV and the IFP gradient are zero in this area. Therefore, the only mechanism of drug delivery into the necrotic core is diffusion through the interstitium, which takes a long time for macromolecules [70]. Due to this reason, as shown in Figure 9a,b, Figure 10a,b and Figure 11a,b, the drug does not reach the necrotic area in the early time after injection. The vessel density in the semi-necrotic region is less than that of the well-vascularized part. Thus, depending on the transport properties of solutes and tumor size, the concentration in the well-vascularized region is higher than the concentration in the semi-necrotic area in all different values of α at some times after injection (Figure 9a, Figure 10a,b and Figure 11a–d).

Despite higher vessel density in the well-vascularized region, the solute concentration is higher in the semi-necrotic area in some αs after a certain amount of time. For example, as shown in Figure 9b (8 h post-injection), the concentration of $F(ab^{\prime })$ in the semi-necrotic area is higher than that of the well-vascularized region in α=27.833, 22.103, 16.122, 9.4795. This phenomenon is due to two reasons. First, when trans-vascular diffusion plays a significant role in the drug delivery mechanism. In this case, more solute transfers to the well-vascularized region compared to the semi-necrotic area because of greater vessel density in the well-vascularized region. After a while, which depends on the normalization intensity, $C_{i}$ becomes greater than $C_{p}$ in the well-vascularized region so, the solute returns to the plasma, and the amount of interstitial concentration starts to decrease. However, $C_{i}$ is still less than $C_{p}$ in the semi-necrotic area at the same timespan frame, and solute diffusion continues from vessels to the interstitium. So, the solute concentration in the semi-necrotic area becomes higher than that of the well-vascularized region at a specified timespan, like what happens in Figure 9b. This time span is dependent on the solute type and tumor size. Second, in later times, the concentration in the semi-necrotic area becomes more than that of the well-vascularized region due to the effect of diffusion through the interstitium to transfer the solute from the zones with high concentration into the necrotic part, such as IgG in $R_{eq}=1.86\;\mathrm{cm}$ and α= 9.4795. It should be noted that once trans-vascular convection becomes the predominant mechanism of solute transport under the influence of reducing the tumor size, normalization, and solute type, the increase in concentration in the semi-necrotic area in comparison to the well-vascularized region occurs only over long periods when the drug is transferred to the necrotic area, such as IgG in $R_{eq}=0.46\;\mathrm{cm}$ and all αs, while in situations that trans-vascular diffusion has a primary role, this phenomenon occurs for both above-mentioned reasons.

According to Table 3, the AAIN affects the parameters of fluid flow and solute transport. These changes influence the various mechanisms of drug delivery, directly or indirectly. Normalization causes the pressure gradient and subsequently non-zero IFV in the inner areas of tumor tissue, as shown in Figure 8. The pressure gradient, or in other words, the difference between IFP and $P_{eff}$, has an effect on the blood vessel source ($\phi_{B}$) and consequently causes convection through the vessel wall (the third term on the right-hand side of Equation (6)). Non-zero IFV activates the convection mechanism in the interstitium in the drug delivery process (second term on the right-hand side of Equation (6)). Drug delivery through the diffusion term from the blood vessel walls (the fourth term on the right-hand side of Equation (6)) changes due to the change in P, $\frac{S}{V}$, and also $\phi_{B}$ caused by normalization. Normalization affects the diffusion in the interstitium (the first term on the right-hand side of Equation (6)) indirectly a long time after injection, which is explained in more detail in the following sections.

As shown in Figure 9a, Figure 10a and Figure 11a, the drug concentration increases at the periphery between tumor and normal tissue suddenly, due to the bump in the IFV profile (steep pressure gradient) in this area. After the early hours, the location of this jump shifts to the normal tissue slowly, which indicates the bulk transport of the drug due to the outflow convection (Figure 9b–d, Figure 10b–d and Figure 11b–d). Normalization reduces the intensity of this jump by modifying the IFV behavior. Additionally, the interval with concentration jump shifts slowly to areas close to the tumor border by normalization because areas with non-zero IFV in the tumor section become wider by normalization, but IFV of normal tissue tends to zero in areas closer to the boundary (Figure 8b). In other words, normalization reduces drug wash-out to the normal tissue. It also decreases the release of tumor growth factors into the surrounding tissue. It is worth mentioning that normalization is considered in the tumor tissue, and normal tissue has the same characteristics for all αs in this study. However, the effect of normalization on the IFV is also evident in normal tissue (Figure 8b) because IFV depends on the pressure gradient, which is affected by normalization even in the normal part of the boundary area (Figure 8a).

It is found that trans-vascular diffusion has a significant role in transferring all three macromolecules into the interstitium other than the areas near the inner boundary in the original size of the tumor by computing the Pe number.

The interstitium concentration of $F(ab^{\prime })$ reaches the plasma concentration faster due to the higher P and lower τ, so $F(ab^{\prime })$ returns to the blood vessels faster. Normalization controls the diffusion rate from or to the blood vessels by decreasing P and $\frac{S}{V}$. $F(ab^{\prime })$ concentration has a better distribution under the vascular normalization after the initial hours. For example, 8 h post-injection (Figure 9b), the distribution of antibody is improved from the point of view of drug exposure amount and uniformity at α=8.5863 and α=9.4795 in comparison to α=27.833 (untreated tumor). After 24 h post-injection (Figure 9c), α=5.9042 and α=6.7988 have better concentration distributions. In fact, at different times, different intensities of normalization can improve drug delivery. This is because there is a trade-off between P, $\frac{S}{V}$ and $C_{p}$ at a specific time so that, if P and $\frac{S}{V}$ have such values that allow trans-vascular diffusion to deliver $F(ab^{\prime })$ into the tissue and return it to the plasma, both in a controlled manner, the distribution of $F(ab^{\prime })$ will be improved. It means that by modifying P and $\frac{S}{V}$ due to the normalization, the solute enters the tissue slowly and the tissue is exposed to the solute for a longer time, unlike rapid solute absorption in untreated tumors. This also causes the clearance behavior to be different in untreated tumors and normalized ones. The solute returns to the plasma faster in the untreated tumor. Additionally, the clearance rate of solute is higher in the untreated tumor compared to the one undergoing anti-angiogenic therapy. Therefore, in general, at a certain time, definite intensities of normalization improve the solute distribution in terms of amount and uniformity.

Normalization causes a better distribution of $F{\left(ab^{\prime }\right)}_{2}$ in areas far from the periphery at 24 and 72 h post-injection. α=22.103 and α=16.122 improve $F{\left(ab^{\prime }\right)}_{2}$ distribution at 24 h post-injection in comparison to the untreated tumor (α=27.833) according to Figure 10c. $F{\left(ab^{\prime }\right)}_{2}$ is distributed in a narrow area of the tumor tissue in the untreated tumor at 72 h post-injection, and different normalization intensities can modify the behavior of $F{\left(ab^{\prime }\right)}_{2}$ distribution (Figure 10d). The improvement of $F{\left(ab^{\prime }\right)}_{2}$ distribution after normalization is due to what is defined in Figure 9.

The concentration of IgG increases slowly due to its transport properties (lower P and $D_{eff}$ and higher $\sigma_{f}$ and τ in comparison to $F{\left(ab^{\prime }\right)}_{}$ and $F{\left(ab^{\prime }\right)}_{2}$). Accordingly, normalization does not have a significant effect on improving the IgG distribution up to 72 h post-injection (Figure 11a–d). However, the influence of normalization is shown in longer times. For example, Figure S1 shows the IgG concentration distribution 12.5 days post-injection. As seen, the concentration distribution of IgG at α=22.103, α=16.122, α=9.4795, and α=8.5863 is better than that of the untreated tumor (α=27.833). In fact, studies should be performed in a convenient time frame to decide about the normalization efficacy and desired normalization intensity, according to the specific characteristics of each drug and tumor size. In addition to what is said about normalization performance in improving $F(ab^{\prime })$ distribution in Figure 9, trans-vascular convection is improved by normalization and contributes to a better IgG distribution as the Pe number is non-zero for IgG, especially in the well-vascularized region. Nevertheless, trans-vascular diffusion is dominant in IgG distribution in the original size according to the Pe number in the interstitium far from the boundary.

Figure S2 shows the concentration distribution of the different macromolecules at final times. According to Figure S2, antibodies reach the necrotic area at longer times, which is called the reservoir phenomenon [18,19]. This phenomenon occurs when, over a long period, the solute reaches the necrotic area through diffusion in the interstitium. At these times, the solute is cleared from the tissue far from the margin by diffusion from the vessels and washed out by convection outflow in the boundary zone. According to Figure S2, it is seen that different macromolecules have different efficient functions. For example, $F(ab^{\prime })$ reaches the necrotic area faster. IgG reaches longer times but has a higher amount.

As mentioned earlier, the solute transfers to the necrotic core only through diffusion in the interstitium. As shown in Figure S2, the amount of solute present in the necrotic area increases by normalization in comparison to the untreated tumor. The normalization intensities that enhance the reservoir effect are different for various solutes. Actually, the intensity of normalization in which the amount of characteristics such as P and $\frac{S}{V}$ is so large that the plasma solute is absorbed into the tissue in the early stages and so small that the material is returned to the blood vessels slower in long times has the better reservoir effect. This phenomenon shows the indirect effect of normalization induced by anti-angiogenic therapy on the diffusion transfer mechanism through the interstitium.

ASCD and DASCD of different macromolecules in different sizes of tumor and various normalization intensities are found. ASCDNE and DASCDNE in the duration of the final third are examined to study the average reservoir behavior distribution (ARBD) and its deviation (DARBD). The following figures show the best normalization intensities in improving the average behavior of macromolecule delivery in different tumor sizes.

**Figure 12:**
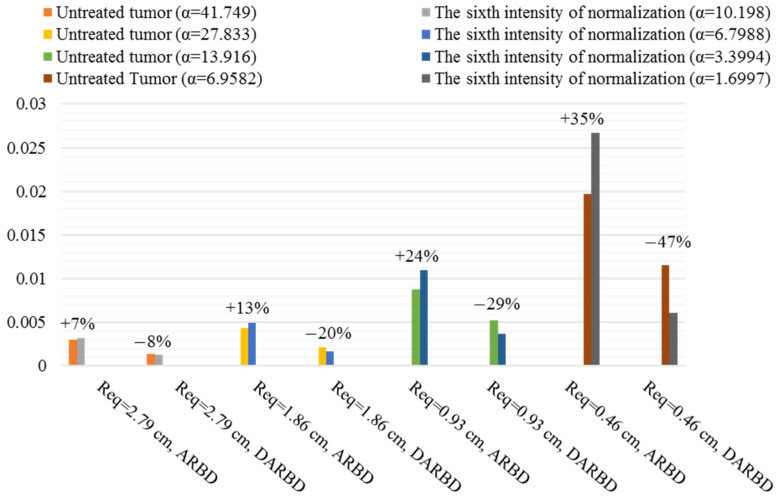
Summary of the positive influence of normalization on ARBD and DARBD of $F(ab^{\prime })$ in different sizes. The percentage of change after applying the AAIN is seen above the bars.

Figure 12 shows the ARBD and DARBD of $F(ab^{\prime })$ in the untreated tumor and the normalized one with an intensity that causes the highest antibody exposure amount and lowest non-uniformity, i.e., the sixth of seven normalization intensities in different sizes. The smaller the tumor size, the greater the increase in ARBD and the greater the decrease in DARBD. The percentage of changes is illustrated in Figure 12. It is found that trans-vascular diffusion has a significant effect on the $F(ab^{\prime })$ transport mechanism in all sizes by calculating the Pe number. Modifying the diffusion from or into the blood vessels due to the normalization improves ARBD and DARBD. Intensifying normalization causes amelioration in reservoir behavior. However, normalization does not have a positive effect if the interstitium properties are similar to the normal tissue.

**Figure 13:**
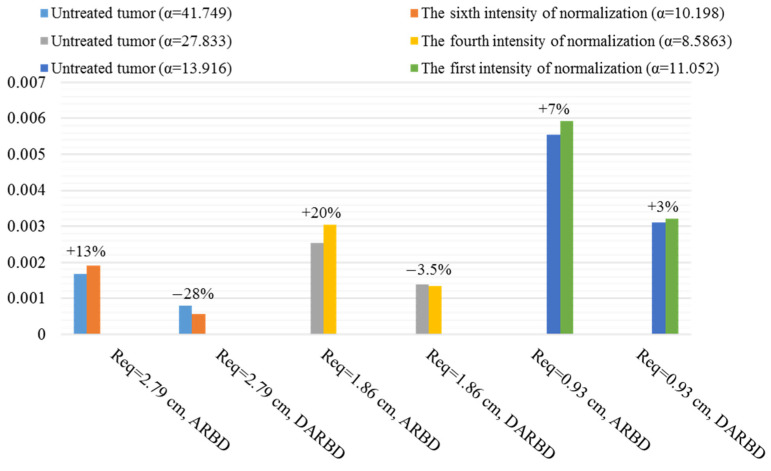
Summary of the positive influence of normalization on ARBD and DARBD of $F{\left(ab^{\prime }\right)}_{2}$ in different sizes. The percentage of change after applying the AAIN is seen above the bars.

**Figure 14:**
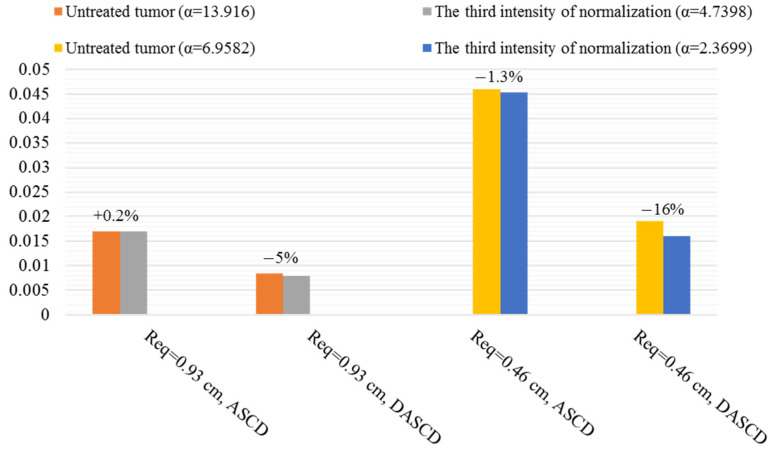
Summary of the positive influence of normalization on ASCD and DASCD of $F{\left(ab^{\prime }\right)}_{2}$ in different sizes. The percentage of change after applying the AAIN is seen above the bars.

Figure 13 and Figure 14 demonstrate the improvements induced by normalization in $F{\left(ab^{\prime }\right)}_{2}$ delivery in different tumor sizes. The percentage of changes induced by normalization is shown in Figure 13 and Figure 14. Minor improvements are achieved in reservoir behavior and solute distribution average behavior once the tumor size decreases to $R_{eq}=0.93\;\mathrm{cm}$. In fact, this size of the tumor in $F{\left(ab^{\prime }\right)}_{2}$ acts as a turning point so that a greater decrease in tumor size to $R_{eq}=0.46\;\mathrm{cm}$ causes amelioration in therapeutic antibody distribution average behavior, as seen in Figure 14, because convection becomes the dominant mechanism in $R_{eq}=0.46\;\mathrm{cm}$ according to the Pe number. Decreasing the tumor size causes the establishment of pressure gradient and subsequently non-zero IFV in more inner areas far from the boundary. This provides the facility for convection improvement induced by normalization to modify the solute delivery, as shown in Figure S3.

Figure S3 illustrates the trans-vascular convection/$C_{p}$ and IFV of $F{\left(ab^{\prime }\right)}_{2}$ in $R_{eq}=0.46\;\mathrm{cm}$ for different normalization intensities in semi-necrotic and well-vascularized regions to show the effect of normalization on convection as the dominant transfer mechanism in this size. There are two intensities with better behavior than others, i.e., α=4.0305 and α=2.3699. The uniformity increase in α=4.0305 and α=2.3699 is 7% and 16%, respectively; however, the ASCD is almost equal to that of the untreated tumor for both αs. Therefore, the best intensity of normalization, in this case, is α=2.3699. Less gradient of trans-vascular convection and IFV in α=2.3699 is responsible for more uniformity of drug distribution.

**Figure 15:**
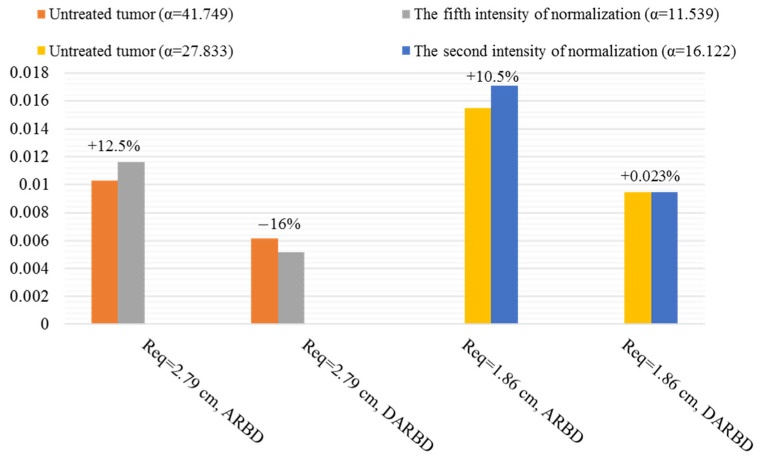
Summary of the positive influence of normalization on ARBD and DARBD of IgG in different sizes. The percentage of change after applying the AAIN is seen above the bars.

**Figure 16:**
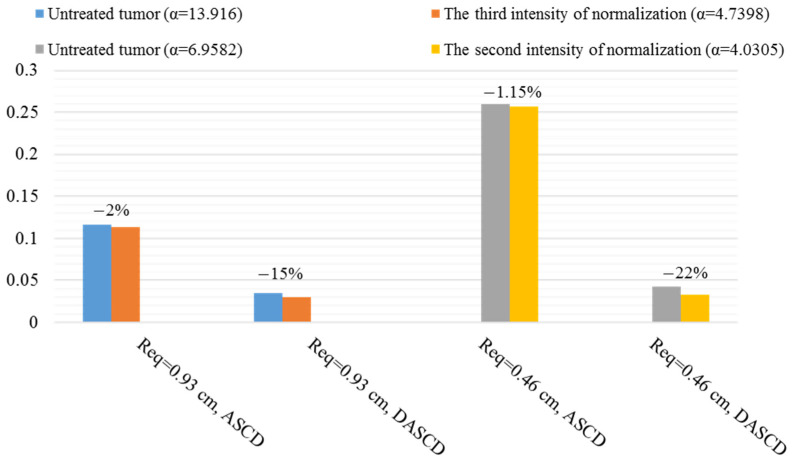
Summary of the positive influence of normalization on ASCD and DASCD of IgG in different sizes. The percentage of change after applying the AAIN is seen above the bars.

The reservoir behavior of IgG is improved in $R_{eq}=2.79\;\mathrm{cm}$ and $R_{eq}=1.86\;\mathrm{cm}$, as seen in Figure 15. Decrease in tumor size to $R_{eq}=0.93\;\mathrm{cm}$ and $R_{eq}=0.46\;\mathrm{cm}$, which causes an enhancement in convection effect, results in better IgG distribution average behavior (Figure 16). This is because of what is explained in the previous paragraph. The Pe number of IgG is more than $F(ab^{\prime })$ and $F{\left(ab^{\prime }\right)}_{2}$ under the same conditions, which indicates more dependence of transport mechanism to convection.

Assuming that the tumor is well-vascularized and homogeneous, the DASCD of IgG is reduced by about 25% in $R_{eq}=0.93\;\mathrm{cm}$. More uniformity in the homogeneous tumor compared to that of this research’s tumor is due to the fact that different parts of real tumors are considered in the present study. In the tumor considered in this research, IFP is uniformly high in the necrotic core, while in the equivalent homogeneous tumor, the pressure gradient and so non-zero IFV are established in whole tumor tissue. This results in convective drug delivery even in the tumor center.

The reservoir effect does not improve in IgG ($R_{eq}=0.93\;\mathrm{cm}$ and $R_{eq}=0.46\;\mathrm{cm}$) and $F{\left(ab^{\prime }\right)}_{2}$ ($R_{eq}=0.46\;\mathrm{cm}$) at long times. This is dependent on the ASCDNE behavior. As an example, Figure S4 shows the ASCDNE of IgG in $R_{eq}=0.46\;\mathrm{cm}$ for untreated tumors and normalized one with α=4.0305. As discussed before, convection is the dominant mechanism of IgG transfer in this size. AAIN establishes the pressure gradient and subsequently causes non-zero IFV in the inner areas close to the necrotic core. Then, more therapeutic agent enters the necrotic core by diffusion through the interstitium in early times in comparison to the untreated tumor. The difference between the solute concentration amount in the necrotic core and viable regions in the normalized tumor becomes more than that of the untreated tumor over time as the solute concentration decreases in the interstitium. Therefore, the antibody diffusion from the necrotic core to the viable regions in the normalized tumor is more intense compared to the untreated tumor. As seen in Figure S4, the ASCDNE is decreased in the normalized tumor with a steeper slop so that it is less than that of the untreated tumor in longer times. Note that in this study, the reservoir behavior is considered in the duration of the final third, in which the ASCDNE of normalized tumor is less than the ASCDNE of the untreated tumor. This is the reason for the lack of improvement in reservoir behavior by normalization when convection is the dominant drug transfer mechanism.

According to the results, it is found that clarification about the efficiency of AAIN is possible by considering the mechanisms affecting solute transfer, which is a parameter that depends on the size of the tumor and the type of drug. $F(ab^{\prime })$ delivery relies heavily on diffusion due to its transport properties. $F(ab^{\prime })$ penetrates the necrotic core better than the two others, and normalization improves this attitude. $F(ab^{\prime })$ administration along with the anti-angiogenic therapy could be used to target the cells of the necrotic area, which have a significant effect on tumor growth. IgG has the potential of exploiting the modification of interstitial fluid flow induced by normalization to improve its delivery because the Pe number is non-zero even in $R_{eq}=2.79\;\mathrm{cm}$. Decreasing the tumor size causes enhancement in convection function in IgG transfer. Subsequently, improvement of interstitial fluid flow behavior induced by normalization increases the quality of IgG distribution in the tumor tissue.

Figure S5 shows the ASCD and DASCD in the untreated tumor and the tumor with characteristics of normal tissue, as aggressive normalization intensity, in different tumor sizes and solute types. Aggressive normalization does not have a constructive effect on drug delivery. This is because the tumor capillary network is like a double-edged sword, which on one hand disrupts efficient drug delivery due to properties such as high complexity and permeability, and on the other hand utilizes characteristics such as high permeability and surface area per unit volume to transfer the therapeutic agents. This reason for the present study’s result has been supported by experimental research [71,72] and was addressed in the literature [17,73]. Moreover, aggressive normalization cannot improve bulk mechanisms of drug delivery, according to Figure 8b and Figure S3.

### 4.3. Limitations and Future Works

In the present study, the effect of normalization induced by anti-angiogenic therapy on drug delivery into the solid tumor was discussed numerically. However, there exist some assumptions and limitations in the current research. The model was performed with a macroscopic point of view, and variations over the microscopic scale were average. Accordingly, for analyzing the function of normalization, the effect of anti-angiogenic therapy on transport properties of the interstitium was considered instead of including the tumor microvascular network by mathematical simulation [26] or image processing [27,74] under the influence of anti-angiogenesis. Another assumption is that the model was assumed to be 2D axisymmetric because there is one image of the tumor. It should be noted that due to the limitations of available laboratory facilities, the present study was not validated experimentally. Then, the predictions derived from the results of this research can be more qualitative than quantitative.

Future plans for the present investigation will be to study the effects of the anti-angiogenic drug in the resolution of the blood vessels and develop a multi-scale model to predict anti-angiogenic therapy in combination with chemotherapy.

## 5. Conclusions

In this research, a numerical approach that couples the mathematical models of interstitial flow and solute transport is developed to provide a detailed study on the role of AAIN in drug delivery into a non-homogeneous solid tumor based on a real tumor image with normal tissue around it. IgG and fragments ($F{\left(ab^{\prime }\right)}_{2}$ and $F(ab^{\prime })$) are considered to evaluate the effect of drug type, and various αs are defined to figure out the influence of normalization intensity. In addition to the original size of the tumor, three other sizes are also considered in analyzing the ASCD and DASCD. The following conclusions are redrawn based on the results:

Normalization causes the reduction in IFP and establishment of the pressure gradient and consequently non-zero IFV in the inner areas of the tumor. The decrease in IFP drop increases by normalization intensification.

The spread of non-zero IFV to the tumor tissue far from the boundary continues as normalization intensifies until a certain extent, after which only the IFV decreases at the tumor margin.

In all tumor sizes, normalization reduces the drug wash-out to the normal tissue by controlling the sudden concentration increase at the periphery between tumor and normal tissue and shifting the areas with concentration jump closer to the tumor.

Normalization can improve the solute concentration distribution in a time-dependent manner. In order to figure out the efficacy of normalization in improving the distribution of therapeutic agents, not only normalization intensity, but also the time after administration must be considered. Thus, the results of this study could be used to exploit AAIN for improving drug distribution regarding its drug-dependent temporal behavior.

According to the ASCD and DASCD bar charts, aggressive normalization may not be efficient. Compared to the untreated tumors, the same degree of normalization of characteristics of the tumor as that of normal tissue results in less solute reaching the tumor.

For exploiting normalization in drug delivery improvement, it is necessary to pay attention to which drug should be used in different tumor sizes. In large sizes, therapeutic agents that rely the most on the trans-vascular diffusion, such as $F(ab^{\prime })$, have a better operation in combination with the anti-angiogenic therapy because of two reasons: (1) these drugs distribute more uniformly in tumor tissue compared to the drugs whose dominant transport mechanism is convection. This is due to that delivery of drugs that depends on convection in large tumor sizes could utilize the improvement of fluid flow behavior induced by normalization only in areas close to the boundary. However, the modification of blood vessel properties under the influence of normalization occurs wherever there is a source. (2) These drugs reach the necrotic area faster, and normalization enhances the reservoir behavior. It is worth mentioning that $F(ab^{\prime })$ returns to the plasma faster due to rapid clearance. The tumor can be more exposed to $F(ab^{\prime })$ by repeated injections. In small sizes, the convective-dependent therapeutic agents such as IgG in combination with anti-angiogenic therapy have more efficiency because the improvement of fluid flow behavior caused by normalization is not limited to the periphery.

There is a relationship between the efficient normalization intensity and the dominant mechanism of drug delivery. The reservoir effect of $F(ab^{\prime })$ benefits from more intense normalization compared to $F{\left(ab^{\prime }\right)}_{2}$ and IgG. Once the convection effect becomes comparable to diffusion, such intensity of normalization improves the ASCD that has the better IFP and IFV distribution.

The results of this research can be used as a guideline for preclinical and clinical studies, which desire to use anti-angiogenic therapy in combination with chemotherapy in solid tumors treatment.

## Figures and Tables

**Table 1:** Variables of Equations (1) and (2).

Variable	Description	Equation	Zone(s) under the Influence of Variable
$\phi_{B}$ ^1,3^	Source term of the fluid flow analysis	$\frac{L_{p}S}{V}(P_{B}-P_{i}-\sigma_{s}(\pi_{B}-\pi_{i}))$	Tumor and Normal tissues
$\phi_{L}$ ^1^	Sink term of the fluid flow analysis	$\frac{L_{pL}S_{L}}{V}(P_{i}-P_{L})$	Normal tissue
$\varphi_{B}$ ^1,2,3^	Source term of the solute transport analysis	$\phi_{B}(1-\sigma_{f})C_{p}+\frac{PS}{V}(C_{P}-C_{i})\frac{Pe}{e^{Pe}-1}$	Tumor and Normal tissues
$\varphi_{L}$	Sink term of the solute transport analysis	$\phi_{L}C_{i}$	Normal tissue

^1^ Descriptions of the parameters of variables are available in Section 2.5. ^2^ Pe is the Peclet number, and its equation is $\frac{\phi_{B}(1-\sigma_{f})V}{PS}$. $C_{P}$ shows the concentration of plasma solute. ^3^ Because the density of blood vessels in different areas of tumor tissue is not the same, $\phi_{B}$ and $\varphi_{B}$ have different values in various parts of the tumor. $\phi_{B}$ and $\varphi_{B}$ are considered to be zero in the necrotic area. According to the study of Lyu et al. [49], the value of $\phi_{B}$ and $\varphi_{B}$ in the semi-necrotic region is considered to be ~0.58 of that of $\phi_{B}$ and $\varphi_{B}$ in the well-vascularized region.

**Table 2:** BCs of fluid flow and solute transport analyses.

Zone	Tumor Center	Inner Boundary	Outer Boundary
Fluid flow	$\nabla P_{i}=0$	$-k_{t}{\nabla P_{i}|}_{R^{-}}=-k_{n}{\nabla P_{i}|}_{R^{+}}$ ^1^ $P_{i}{|}_{R^{-}}=P_{i}{|}_{R^{+}}$ ^1^	$P_{i}=P_{sur}$
Solute transport	$D_{eff}\nabla C_{i}+{\vec{V}}_{i}C_{i}=0$	$(D_{eff}{}^{t}\nabla C_{i}+{\vec{V}}_{i}C_{i}){|}_{R^{-}}=(D_{eff}{}^{n}\nabla C_{i}+{\vec{V}}_{i}C_{i}){|}_{R^{+}}$ ^1^ $C_{i}{|}_{R^{-}}=C_{i}{|}_{R^{+}}$ ^1^	$-n\cdot \nabla C_{i}=0$ ^2^

^1^ $R^{-}$ and $R^{+}$ show the radius of the tumor and normal tissues at the inner boundary, respectively. $k_{t}$ and $k_{n}$ are hydraulic conductivity of the interstitium in tumor and normal tissues [15]. ^2^ n depicts the normal vector.

**Table 3:** Baseline value of fluid flow and solute transport properties.

Parameter	Description	Normal Tissue	Normalized Tissue	Tumor Tissue	Reference(s)
$L_{p}\;(\frac{\mathrm{cm}}{s\;\mathrm{mmHg}})$	Hydraulic conductivity of the microvascular wall	$3.6\times 10^{-8}$	$5.6\times 10^{-8}$	$2.8\times 10^{-7}$	[18,40,53]
$k\;(\frac{{\mathrm{cm}}^{2}}{s\;\mathrm{mmHg}})$	Hydraulic conductivity of the interstitium	$2.5\times 10^{-7}$	$2.5\times 10^{-7}$	$2.5\times 10^{-7}$	[40]
$\frac{S}{V}\;(\frac{{\mathrm{cm}}^{2}}{{\mathrm{cm}}^{3}})$	Surface area of vessel wall per unit volume of tissue	70	116 ^a^	200	[22,23,55,56]
$P_{B}\left(\mathrm{mmHg}\right)$	Vascular pressure	15.6	15.6	15.6	[22,23]
$\pi_{B}(\mathrm{mmHg})$	Osmotic pressure of the plasma	20	19.2	19.8	[40]
$\pi_{i}(\mathrm{mmHg})$	Osmotic pressure of the interstitial fluid	10	15.1	17.3	[40]
$\sigma_{s}$	Average osmotic reflection coefficient for plasma proteins	0.91	$2.1\times 10^{-3}$	$8.7\times 10^{-5}$	[58] and calculated based on [59,60]
$P_{L}(\mathrm{mmHg})$	Hydrostatic pressure of the lymphatics	0	-	-	[66]
$\frac{L_{PL}S_{L}}{V}(\frac{1}{s\;\mathrm{mmHg}})$	Product of hydraulic conductivity of the lymphatic wall and surface area of lymphatic wall per unit volume of tissue	$1.33\times 10^{-5}$	-	-	[66]
$\sigma_{f}$	Osmotic filtration reflection coefficient	$F{\left(ab^{\prime }\right)}_{2}:0.9$	$F{\left(ab^{\prime }\right)}_{2}:2.06\times 10^{-3}$	$F{\left(ab^{\prime }\right)}_{2}:8.41\times 10^{-5}$	[62] and calculated based on [59,60]
		$F(ab^{\prime }):0.5$	$F(ab^{\prime }):7.33\times 10^{-4}$	$F(ab^{\prime }):2.96\times 10^{-5}$	
		IgG:0.95	$IgG:2.43443\times 10^{-3}$	$IgG:9.9358\times 10^{-5}$	
$D_{eff}(\frac{{\mathrm{cm}}^{2}}{s})$	Effective diffusion coefficient	$F{\left(ab^{\prime }\right)}_{2}:0.16\times 10^{-8}$	$F{\left(ab^{\prime }\right)}_{2}:2\times 10^{-8}$	$F{\left(ab^{\prime }\right)}_{2}:2\times 10^{-8}$	[63]
		$F(ab^{\prime }):1.2\times 10^{-8}$	$F(ab^{\prime }):4.4\times 10^{-8}$	$F(ab^{\prime }):4.4\times 10^{-8}$	
		$IgG:0.048\times 10^{-8}$	$IgG:1.3\times 10^{-8}$	$IgG:1.3\times 10^{-8}$	
$P(\frac{\mathrm{cm}}{s})$	Microvessel permeability coefficient	$F{\left(ab^{\prime }\right)}_{2}:2.2\times 10^{-8}$	$F{\left(ab^{\prime }\right)}_{2}:9.54\times 10^{-8}$	$F{\left(ab^{\prime }\right)}_{2}:17.3\times 10^{-8}$	[23,63,65]
		$F(ab^{\prime }):19.1\times 10^{-8}$	$F(ab^{\prime }):82.2\times 10^{-8}$	$F(ab^{\prime }):149\times 10^{-8}$	
		$IgG:0.73\times 10^{-8}$	$IgG:3.16138\times 10^{-8}$	$IgG:5.73\times 10^{-8}$	
τ(h) ^b^	Drug half-life in plasma	$F{\left(ab^{\prime }\right)}_{2}=4.2$	$F{\left(ab^{\prime }\right)}_{2}=4.2$	$F{\left(ab^{\prime }\right)}_{2}=4.2$	[18]
		$F(ab^{\prime })=2$	$F(ab^{\prime })=2$	$F(ab^{\prime })=2$	
		IgG=72	IgG=72	IgG=72	

^a^$\frac{S}{V}\;$ is assumed to decrease 42% after normalization in comparison to the pretreatment level (tumor tissue). This value is considered as an average value of the decrease in permeability surface area product reported in studies of [55,56]. ^b^ τ is the drug half-life and introduced in bolus injection.

## Data Availability

The data presented in this research are available on request from the corresponding author.

## Supplementary Materials

The following are available online at https://www.mdpi.com/article/10.3390/pharmaceutics14020363/s1; Figure S1: Distribution of IgG concentration along line 1, 12.5 days post-injection; Figure S2: Distribution of therapeutic antibodies concentration along line 1. (a) $F(ab^{\prime })$, 8.8 days post-injection. (b) $F{\left(ab^{\prime }\right)}_{2}$, 32.3 days post-injection. (c) IgG, 89.9 days post-injection; Figure S3: (a) Trans-vascular convection/ of $F{\left(ab^{\prime }\right)}_{2}$ in $R_{eq}=0.46\;\mathrm{cm}$. (b) IFV in $R_{eq}=0.46\;\mathrm{cm}$; Figure S4: ASCDNE of IgG in $R_{eq}=0.46\;\mathrm{cm}$ for untreated tumor and normalized one with α=4.0305; Figure S5: ASCD and DASCD in the untreated tumor and the tumor with aggressive normalization. (a) $R_{eq}=2.79\;\mathrm{cm}$ and $R_{eq}=1.86\;\mathrm{cm}$. (b) $R_{eq}=0.93\;\mathrm{cm}$ and $R_{eq}=0.46\;\mathrm{cm}$.
