# Supplementary material for: Numerical Investigation on the Anti-Angiogenic Therapy-Induced Normalization in Solid Tumors
Source: Pharmaceutics. 2022 Feb 5;14(2):363. doi: 10.3390/pharmaceutics14020363 (PMC8877966; doi:10.3390/pharmaceutics14020363)
Supplement: Supplementary file 1 [file pharmaceutics-14-00363-s001.zip › pharmaceutics-1528923-supplementary.pdf]

pharmaceutics

Supplementary Materials: Numerical Investigation on the Anti-Angiogenic Therapy-Induced Normalization in Solid Tumors

Mahya Mohammadi [1,2], Cyrus Aghanajafi [1], M. Soltani [1,3,4,5,*] and Kaamran Raahemifar [6,7,8]

ASCD and DASCD Definition

In the present study, average solute concentration distribution (ASCD) and deviation from the average solute concentration distribution (DASCD) are defined to demonstrate the average amount of drug exposure and the non-uniformity of its distribution, respectively. The formulation of ASCD and DASCD are as follows;

$$ASCD : \frac{1}{C_0 T}\int_0^T \left\langle C_i(t) \right\rangle_{\Omega_t} dt \tag{S1}$$

$$DASCD : \frac{1}{C_0 T}\int_0^T \left\langle (C_i(t) - \left\langle C_i(t) \right\rangle_{\Omega_t})^2 \right\rangle_{\Omega_t}^{\frac{1}{2}} dt \tag{S2}$$

where C_0 shows the initial concentration of solute, which is considered to be $1\,mol/m^3$ in this study. T is the final time of the simulation. $\left\langle Character \right\rangle_{\Omega_t} = \frac{1}{V_t}\int_{\Omega_t} (character) d^3r$ shows the average of a character over the tumor zone, in which V_t is the tumor volume.

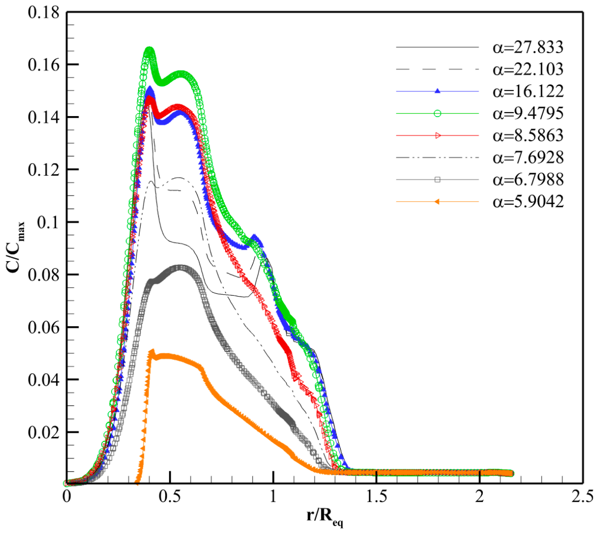

Figure S1. Distribution of IgG concentration along line 1, 12.5 days post-injection.

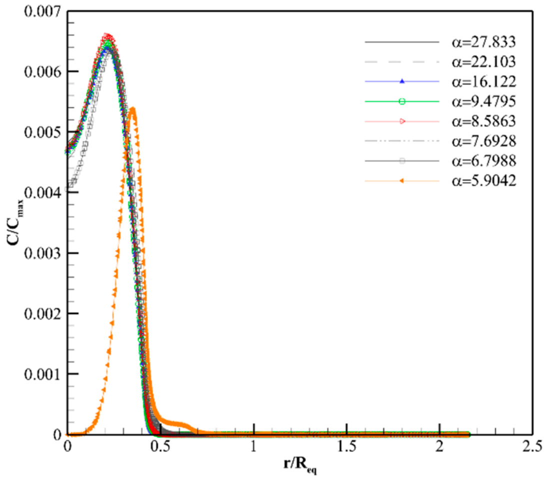

(a)

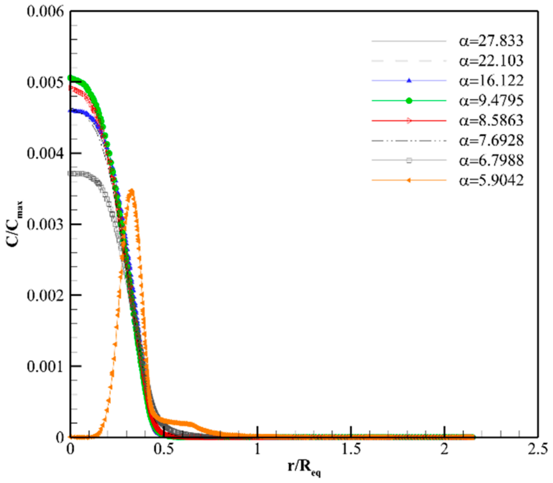

(b)

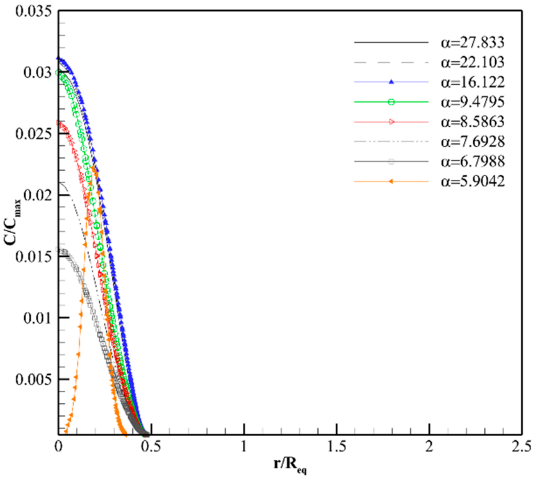

(c)

Figure S2. Distribution of therapeutic antibodies concentration along line 1. (**a**) $F(ab')$, 8.8 days post-injection. (**b**) $F(ab')_2$, 32.3 days post-injection. (**c**) IgG, 89.9 days post-injection.

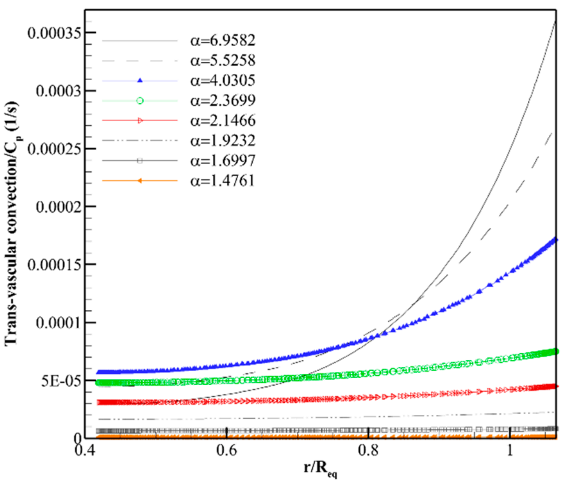

(a)

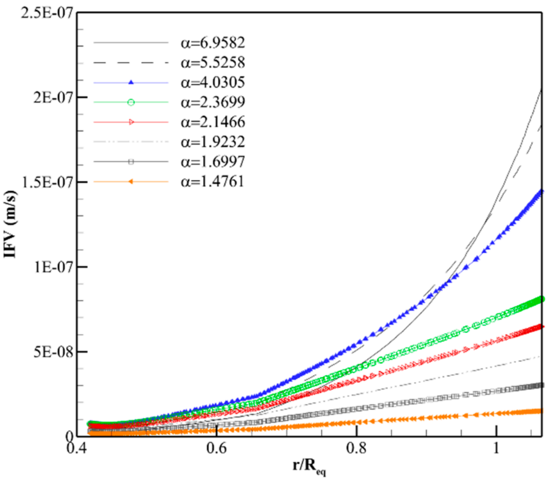

(b)

Figure S3. (**a**) Trans-vascular convection/ C_p of $F(ab')_2$ in $R_{eq} = 0.46\,cm$. (**b**) IFV in $R_{eq} = 0.46\,cm$.

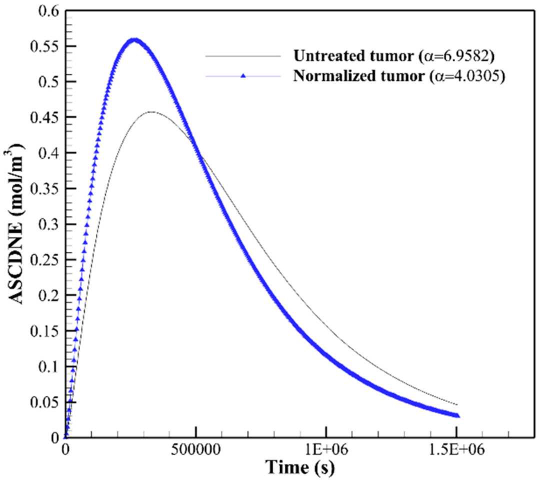

Figure S4. ASCDNE of IgG in $R_{eq} = 0.46\,cm$ for untreated tumor and normalized one with $\alpha = 4.0305$.

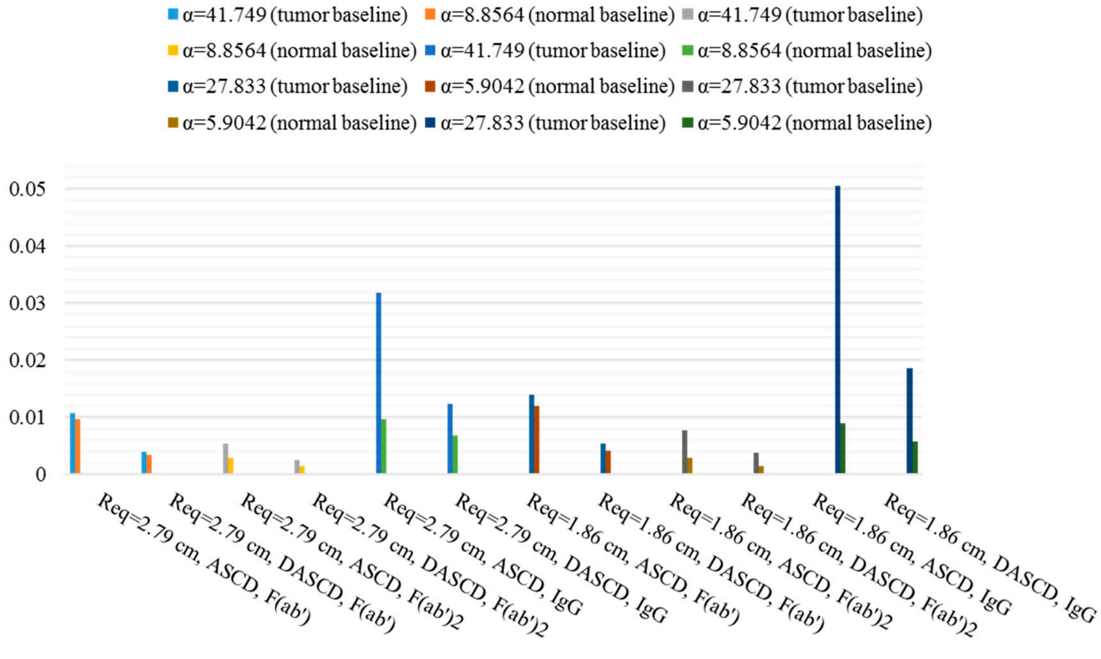

(a)

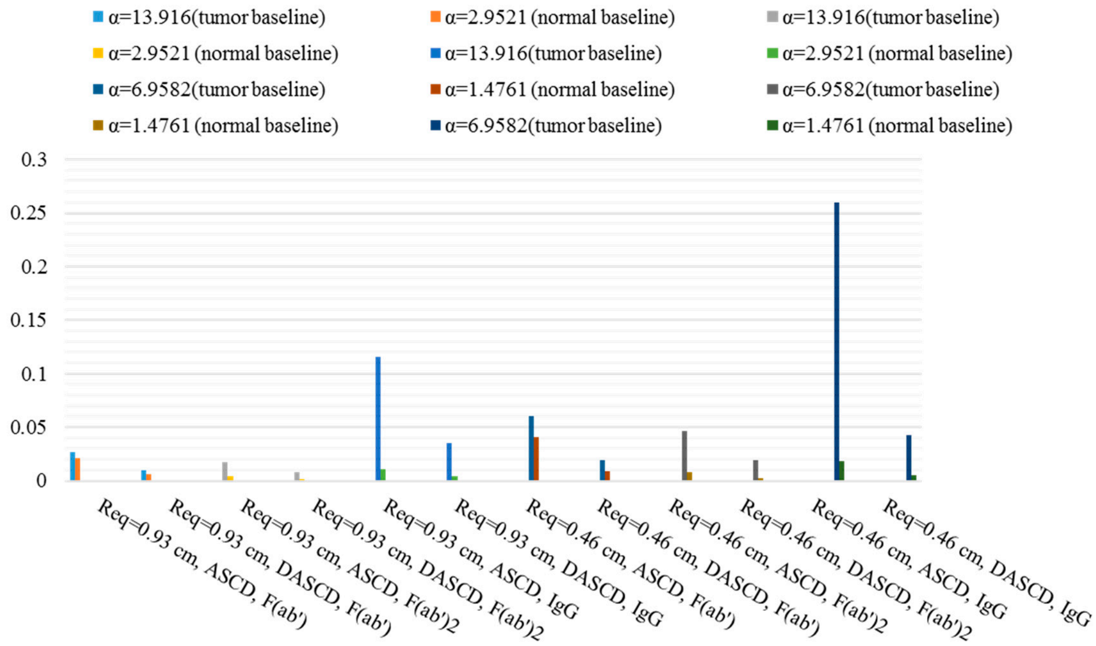

(b)

Figure S5. ASCD and DASCD in the untreated tumor and the tumor with aggressive normalization. (a) $R_{eq} = 2.79\,cm$ and $R_{eq} = 1.86\,cm$. (b) $R_{eq} = 0.93\,cm$ and $R_{eq} = 0.46\,cm$.
